# An emerging *Platynota* sp. (Lepidoptera: Tortricidae) infesting blueberry (*Vaccinium corymbosum*) in the central coast of Peru

**DOI:** 10.3389/finsc.2025.1593907

**Published:** 2025-06-13

**Authors:** Ricardo Velasquez, Ana Maria Leiva, Alejandra Gil-Ordóñez, Lady Susan Perez-Fuentes, Viviana Domínguez, Wilmer J. Cuellar

**Affiliations:** ^1^ Laboratorio de Entomología y Manejo Integrado de Plagas, Instituto Nacional de Innovación Agraria (INIA), Lima, Peru; ^2^ Virology and Crop Protection Research Area, Cassava, Program, Crops for Nutrition and Health, International Center for Tropical Agriculture (CIAT), The Americas Hub, Palmira, Colombia

**Keywords:** emerging pests, mitogenome, diagnostics, taxonomy, biological cycle

## Abstract

Blueberry cultivation has recently become a rapidly expanding export industry in Peru. With few to no official records of phytosanitary problems up to date. Nevertheless, as observed in other major blueberry producer countries, pests occurrences have been already reported. This study presents a comprehensive biological and molecular characterization of a novel blueberry pest, identifying it as a member of the Tortricidae family in the genus *Platynota*. The insect’s average life cycle was determined to be 46.3 days for males and 48.6 days for females, with the larval stage being the longest (25.4 days on average), and the most destructive due to its feeding behavior, which significantly damages buds and fruits. Morphological analysis of the genitalia, along with a comparison of its complete mitochondrial DNA, further supports the conclusion that this pest is a new species. These findings represent the first report of a tortricid pest affecting blueberries in Peru and offer crucial insights for developing effective pest management strategies, contributing to the sustainable growth of blueberry production and exports in the region.

## 1 Introduction

Blueberries (*Vaccinium corymbosum*) have emerged as a high-value crop in the international market, mainly due to their exceptional nutritional profile and high antioxidant content. Peru has positioned itself as a key global supplier, cultivating 16,536 hectares of blueberries, making it the largest producer in Latin America and the third largest worldwide, after Canada and the United States (1). The industry’s rapid expansion has been driven by the introduction of new cultivars specifically adapted to Peru’s warmer climate, enhancing both yield and fruit quality (2). In response to rising global demand, blueberry cultivation in the Lima region alone grew from 120 hectares in 2016 to 1,453 hectares within six years. During the 2021–2022 season, Peru produced approximately 220,000 tons of blueberries (3), successfully expanding exports to the Asian market (4, 5). This remarkable growth underscores Peru’s increasing role in the global blueberry industry and highlights the country’s potential for further market diversification.

A major limitation in large-scale blueberry production is pest infestations, which impact both yield and product quality. To date, no pests have been officially reported on blueberries in Peru. However, in other countries, members of the family Tortricidae have been documented affecting this crop (6, 7). This family is one of the most diverse groups of Lepidoptera, comprising approximately 10,000 species across three subfamilies (Chlidanotinae, Tortricinae, and Olethreutinae). In the Neotropical region, tortricid surveys have been conducted in countries such as the Dominican Republic, Venezuela, Chile, Ecuador, Colombia, and Peru (8–10). Several species within this family have been reported in fruits, particularly berries. Despite the high diversity of tortricids associated with berries and the economic importance of these crops, knowledge of their interactions with host plants remains limited (11, 12). It is also noteworthy that besides the cosmopolitan nature of the family Tortricidae, members of genus *Platynota* Clemens, 1860, are restricted to the Western Hemisphere (9).

Molecular techniques have revolutionized species identification, particularly in molecular and precision entomology. Mitochondrial DNA (mtDNA) exhibits low recombination rates and contains hypervariable regions flanked by highly conserved sequences (13, 14). This unique structure facilitates the differentiation of closely related species while enabling the design of universal primers for broad taxonomic applications (15, 16). Among mitochondrial markers, the cytochrome oxidase I (mtCOI) gene is widely used for species identification. It serves as a basis for DNA barcoding, a method proposed by Hebert in 2003 (17), which provides a rapid and accurate approach to species discrimination (18). Beyond single-gene barcoding, complete mitochondrial genomes provide higher taxonomic resolution by incorporating additional genetic information. In insects, their small size comprising 13 protein-coding genes, 22 transfer RNA genes, and two ribosomal RNA (rRNA) genes (19), coupled with high copy numbers, makes them a powerful tool for genomic studies (20, 21). Moreover, advances in sequencing technologies have further expanded access to whole mitochondrial genome (mitogenome) data, facilitating phylogenetic and evolutionary analyses (14, 22).

In Peru, where blueberry cultivation is rapidly expanding, few official reports exist of insect pests affecting this crop. We present here the results of a recent survey carried out in 2022 in collaboration with the National Institute of Agricultural Innovation (INIA) of Peru in the North of Lima, with the goal to identify emerging pests of blueberry.

## 2 Materials and methods

### 2.1 Study area

We conducted the pest damage on blueberry in the Integrated Pest Management (IPM) Entomology Laboratory of INIA located at the Donoso Agrarian Experimental Station in Huaral, Peru (11°52’ N, 77°23’ E) at an altitude of 180 m.a.s.l. The blueberry crop was cultivated using a high-bed (ridge) system, with beds raised 60 cm above ground level. The plants were grown in pot bags filled with substrate, spaced 0.5 meters apart within rows, and arranged in rows with 2-meter spacing.

### 2.2 Damage assessment

To assess pest damage, periodic evaluations were carried out on 20 plants in three experimental fields at three phenological stages of the blueberry crop. These assessments focused on the presence of pests on leaves, shoots, and fruits, providing a comprehensive analysis of pest-related damage throughout the different growth phases of the crop. At the budding stage, the number of affected and healthy shoots and leaves was recorded to calculate the percentage of damage. Similar evaluations were conducted at the flowering stage (affected and healthy flowers) and fruiting stage (damaged fruits during development and at five harvests). Percentage damage was calculated for each stage, and mean values across replicates were reported. Standard deviations (sd) were calculated using R software (v4.3.0). Data were presented as mean ± sd.

### 2.3 Taxonomical and biological characterization

Larvae collected in the field were reared under laboratory conditions and two adult specimens (one male and one female) were selected for further analysis. The specimens were sent to the Museo de Entomología Klaus Raven Büller at Universidad Nacional Agraria La Molina, Lima, Peru (MEKRB) for morphological identification based on wings and genitalia characteristics. Genital structures were examined through morphological comparisons and identified using taxonomic keys (9, 23). Digital images were taken using a Canon S50 camera mounted on a Leica MZ6 stereomicroscope.

To study the biological cycle of the insect, specimens were collected at various developmental stages (adult, egg, larva, and pupa). These specimens were reared in the laboratory under controlled conditions temperature of 25 ± 2°C, relative humidity RH of 70 ± 10%, and 14 h photoperiod. The duration of each developmental stage was evaluated by monitoring 10 individuals across three experimental replicates. Larvae were individually maintained in Petri dishes and developmental progress was recorded at daily intervals. The onset of larval development was marked by egg hatching and the emergence of the first instar (L1). Subsequent instars were identified by observing the exuviae and cephalic capsules, which served as indicators of transitions between stages until the final molt. In addition, body length was measured using an ocular micrometer mounted on a stereoscopic microscope. After emergence, adult *Platynota* sp. individuals were classified at the genus level based on wing size and coloration. Observations were conducted on 10 pairs of individuals for each species. Daily assessments included longevity, fecundity, pre-oviposition period, oviposition period, and sex ratio. The pre-oviposition period was defined as the number of days between adult emergence and the deposition of the first egg. Females were observed daily to record the onset and duration of egg laying, allowing the estimation of the oviposition period, total oviposition duration, and daily fecundity.

### 2.4 Molecular characterization

For molecular characterization, insects were preserved in 70% ethanol and stored at 4°C. After ethanol removal, samples were placed directly into PCR tubes (24). Each insect underwent three methodological replicates. DNA amplification targeted the mtCOI region of ~700 base pairs (bp) using universal primers LCO1490 (5′-GGTCAACAAATCATAAGATATTGG-3′) and HCO2198 (5′-TAACTTCAGGGTGACCAAAAAATCA-3′) (25). The PCR mix was prepared using 2 μl of DNA, 12.5 μl of GoTaq Green Master Mix 2X (Promega, USA), 0.5 μl of each primer (10 μmol) and completed with nuclease free water to a final volume of 25 μl. PCR was performed in a MiniAmp™ thermal cycler with negative controls included in all runs, using the following program: initial denaturation for 40 s at 95°C, followed by 35 cycles of denaturation for 40 s at 95°C, annealing for 40 s at 54°C and extension for 60 s at 72°C, and a final extension for 10 min at 72°C. PCR products were visualized by 1% agarose gel electrophoresis stained with SYBR^®^ Safe DNA gel stain 10,000 X (Invitrogen, USA).

DNA sequence analysis was carried out to improve taxonomic resolution and classification accuracy. Total DNA was isolated from the head of a field-collected larva (5^th^ instar) using the Dellaporta protocol (26). DNA concentration and quality were measured using a Thermo Scientific NanoDrop 2000 spectrophotometer (260/280 and 230/260 nm), and DNA integrity was assessed by 1% agarose gel electrophoresis stained with SYBR^®^ Safe DNA gel stain 10,000 X (Invitrogen, USA). Good-quality DNA was then used for long-read sequencing.

### 2.5 Sequencing and bioinformatics analysis

The PCR library was prepared using Oxford Nanopore Technologies (ONT) sequencing (SQK-LSK110) and barcode kits (EXP-NBD196), following manufacturer’s protocols. The final library was loaded onto a Flongle flow cell (FLO-FLG110) and run for 24 h. A total DNA library was prepared with the same sequencing kit, loaded onto a R9.4.1 flow cell (FLO-MIN106) and run for 72 h. Sequencing was conducted on the MinION device (Oxford Nanopore Technologies, UK) using the ONT MinKNOW Software. Basecalling was performed with Guppy v6.4.2 in high-accuracy mode (hac) on the raw sequence data, and only high-quality sequences with a Phred score greater than 10 were used for the analysis.

The consensus sequence of each PCR product was assembled with Amplicon_sorter (27) and refined by aligning raw reads to the consensus with Minimap2 v2.11 (28). Subsequently, SAM-BAM conversion was performed with SAMtools v1.3.1 (29). Polishing was conducted with Pilon v1.24 and Medaka v1.2.4, and coverage was assessed using Qualimap v2.2.2 (30). The Barcode of Life Data System (BOLD) identification engine accessed through the BOLD Systems V3 website (31) was used to identify the sequences. This tool allowed us to compare our sequences against the entire International Barcode of Life (iBOL) database, ensuring a comprehensive taxonomic assessment. We selected every mtCOI barcode record with stringent selection parameters, including species-level barcode records from the iBOL database. Only sequences with confirmed species-level identification and a minimum length of 500 bp were considered. The identification engine generated a list of up to 100 closest matches, from which we selected the top 20 records with the highest percentage of identity and bp overlap for further analysis. In addition to BOLD, sequence identification was performed using BLASTn (e-value < 0.05) against the NCBI non-redundant (nr) database to further validate the taxonomic placement. For the phylogenetic analysis, we used 18 sequences obtained from GenBank, including 17 sequences from the subfamily Tortricinae (representing the tribes Sparganothini, Cochylini, and Archipini), which were identified as the closest specimens based on BOLD data, and one sequence from *Brachycentrus kozlovi* (Trichoptera: Phryganeoidea) as an outgroup. The analysis was performed using the Neighbor-Joining (NJ) method in MEGA 11 (32), with 1,000 bootstrap replicates to assess the robustness of the tree topology and provide strong support for most nodes.

The DNA library was assembled using Flye v2.9 (33). The mitochondrial contig was identified assessing the presence of *cox1*, *nad1* and *atp6* genes by BLASTn (e-value < 1e−25). Full annotation was performed with MITOS using Invertebrate Mitochondrial genetic code (RefSeq 89 Metazoan) (34) and coverage was calculated using Qualimap v2.2.2 (30). The mitogenomic features of *Platynota* sp. Per1 were compared with mitogenomes of 22 other tortricid species available in GenBank, using BBmap v38.18 (35). Among these, three species belonged to the tribe Cochylini, one to Cnephasini, three to Tortricini, thirteen to Archipini, and one to Ceracini. No available sequence data were found for the tribe Sparganothini. *Eogystia hippophaecolus* and *Zeuzera multistrigata* were selected as outgroup species. Nucleotide sequences were aligned using MAFFT v7.526 on the Galaxy platform (https://usegalaxy.eu/, accessed on 24 February 2025). Phylogenetic reconstruction was performed using the Maximum Likelihood (ML) method in IQ-TREE (http://www.iqtree.org/, accessed on 24 February 2025), employing the General Time Reversible model with a gamma distribution (GTR+G) and bootstrap support based on 1,000 replicates.

## 3 Results

### 3.1 Pest damage on blueberry crops

**Figure 1:**
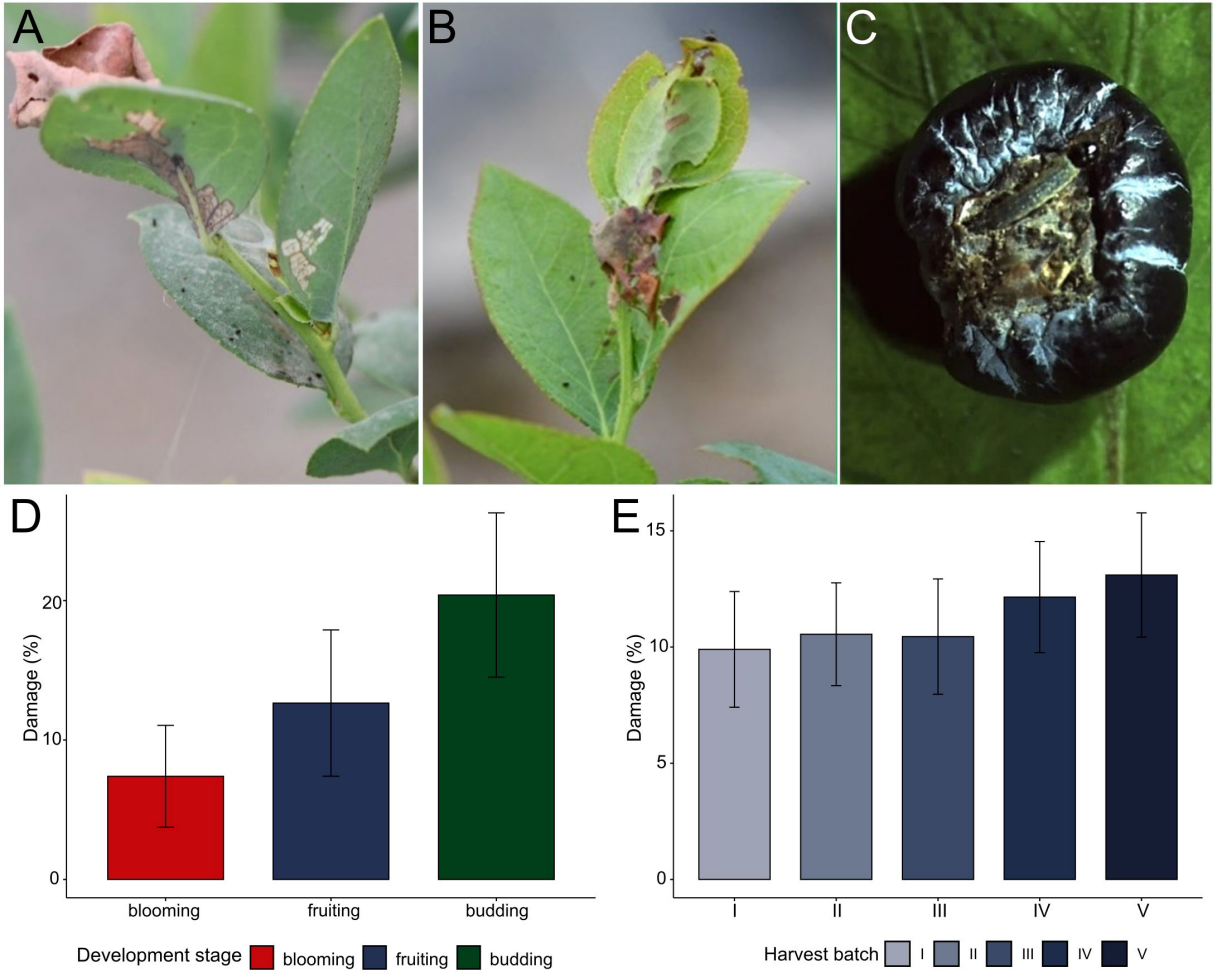
Quantification of damage caused by *Patynota* sp. Per1 to blueberries in Huaral Province, Peru. **(A)** Leaf damage. **(B)** Buds damage. **(C)** Immature fruit damage. **(D)** Percentage of damage caused by *Platynota* sp. at three critical stages of blueberry plant development: budding (green), fruiting (purple) and blooming (red). **(E)** Percentage of blueberry fruist damage caused by *Platynota* sp. Per1 in 5 harvest batches (purple gradient).

The leaf-rolling larva was identified as a significant pest affecting blueberry crops throughout the vegetative, flowering, and fruiting stages. Larval damage was characterized by surface scraping on leaves and buds, leading to irregular development patterns and reduced photosynthetic capacity. Additionally, larvae produced silk threads to bind leaves together, creating protective shelters that facilitated pupation and increased survival rates. The most severe damage was observed on the fruits, where larvae penetrated the berry skin, creating entry points for secondary fungal and bacterial infections, and causing premature rotting and detachment from the plant (**Figure 1**).

Damage assessments conducted across three key developmental stages of blueberries (budding, flowering, and fruiting) revealed the highest damage occurring during the budding stage, with an average of 20.4 ± 5.9%. This was followed by the fruiting stage, where damage averaged 12.6 ± 5.2%. The lowest damage was recorded during the flowering stage, with an average of 7.4 ± 3.6% (**Figure 1D**, **Supplementary Table 1, Sheet 1**). Additionally, damage levels varied throughout the harvest season, ranging from a minimum of 9.9 ± 2.5% in the early harvests to a peak of 13.1 ± 2.7% in the final harvests (**Figure 1E**, **Supplementary Table 1, Sheet 2**). These findings underscore the progressive increase in pest damage over time and emphasize the need for targeted early management during critical crop development periods.

### 3.2 Wing patterns and genital morphology

**Figure 2:**
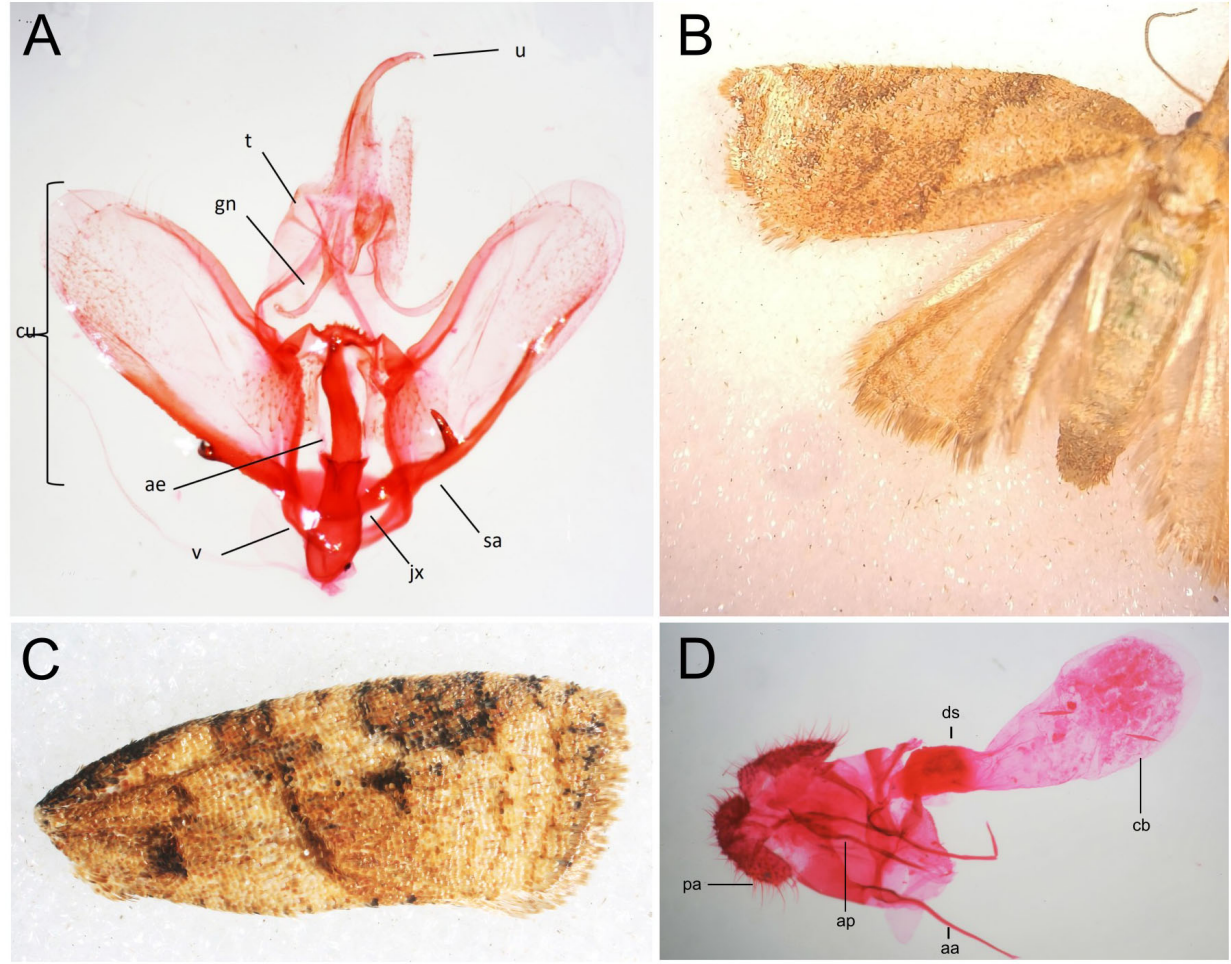
**(A)** Genitalia of a male *Platynota* sp. Per1. ae, aedagus; cu, cucullus; gn, gnatos; jx, juxta; sa, sacculus; u, uncus; v, vinculum. **(B)** Wings of the female. **(C)** Forewing of the male, showing the color pattern of *Platynota* sp. Per1. **(D)** Genitalia of a female *Platynota* sp. Per1. aa, anterior process; ap, posterior process; cb, body of the bursa; ds, seminal duct; pa, anal papilla.

Wing patterns and adult morphology of moths exhibited a characteristic brown and ochre coloration interspersed with dark oblique bands (**Figures 2B, C**). The forewings were slightly elongated, with a curved termen and a reticulated scale pattern. The hindwings were broader, paler, and displayed a translucent appearance with a well-defined fringe along the posterior margin (**Figure 2B**). Genitalia morphology in males exhibited a broad, membranous, and slightly asymmetrical valvae, with fine setae along the margins. The uncus was hook-shaped and well-sclerotized, providing structural support. The phallus was elongated and slightly curved, with a visible vesica, while the transtilla formed a reinforcing structure connecting the valvae. In females, the genitalia featured a well-developed and sclerotized sterigma structured around the genital opening (**Figure 2D**). The ductus bursae were elongated and membranous, leading to large, rounded corpus bursae containing visible signa. The ovipositor lobes were setose, facilitating egg deposition. These are characteristic traits of the *Platynota* genus (**Figure 2A**).

Based on the above-described morphological characteristics, specimens collected from affected *V. corymbosum* plants were compared with available *Platynota* morphological information including members of species *P. rostrana*, *P. flavedana* and *P. sultana* and subsequently were classified as members of the family Tortricidae, genus *Platynota*. Although closer examination of external and genital structures revealed distinct diagnostic features, it was not possible, using the available taxonomy keys (9, 23), to classify these *Platynota* specimen Per1 to the species level.

### 3.3 Biological cycle

**Figure 3:**
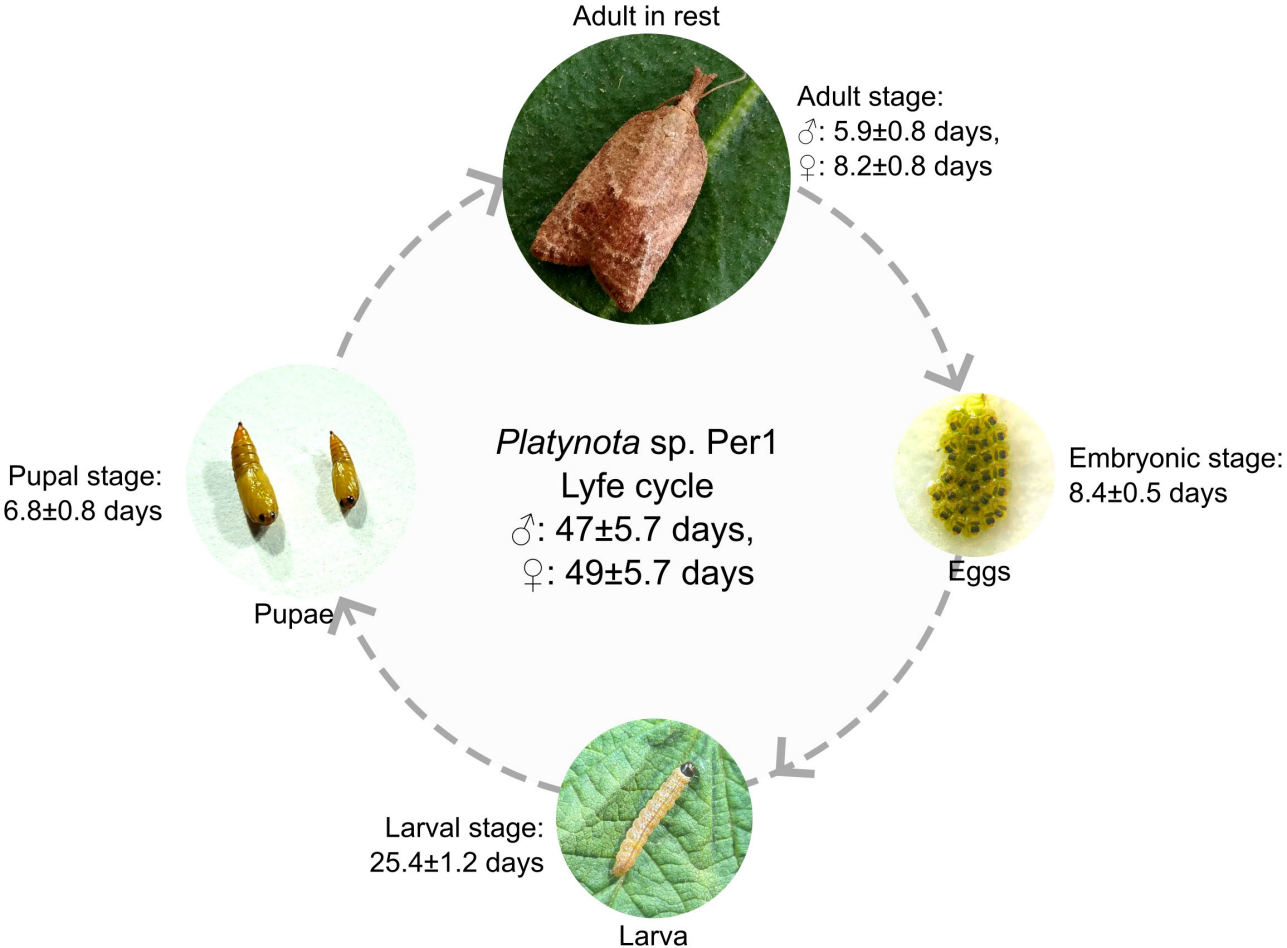
Biological cycle of *Platynota* sp. Per1. The graph shows the different developmental stages described in this work. The insect completes its cycle in less than 50 days.

The life cycle of *Platynota* sp. Per1 was completed in approximately 47 ± 5.7 days for males and 49 ± 5.7 days for females, under laboratory conditions. The embryonic stage lasted 8.4 ± 0.5 days, followed by a larval stage (I, II, III, IV, V, VI) of 25.4 ± 1.2 days, during which the larvae exhibited leaf rolling behavior and actively fed on blueberry foliage. Pupation occurred after larval development, lasting 6.8 ± 0.8 days, resulting in the emergence of the adult. Adult longevity varied between sexes: males survived 5.9 ± 0.8 days and females 8.2 ± 0.8 days. Reproductive parameters we re as follows: pre-oviposition period of 1.6 ± 0.5 days and an oviposition period of 8.4 ± 0.5 days; females laid an average of 169.1 ± 37.6 eggs Regarding morphological dimensions, pupal body length and head area grew from 2.0 ± 0.1 and 0.3 ± 0.0 mm, to 14.4 ± 0.6 and 1.2 ± 0.0 mm between the first and fifth instars, respectively. Pupae showed an average length of 6.9 ± 0.3 mm and a head area of 2.1 ± 0.1 mm. In adults, body size reached a length of 9.8 ± 0.5 mm, with a head width and wing extension of 1.1 ± 0.1 and 17.1 ± 0.8 mm, respectively (**Figure 3**, **Table 1**).

**Table 1:** Summary of biological and morphological features of *Platynota* sp. Per1 under laboratory conditions.

Biological Parameters	Traits	Min	Max	Mean	sd
Embrionic/Eggs (days)		8	9	8.4	0.5
Larval (days)		24	27	25.4	1.2
Pupa (days)		6	8	6.8	0.8
Pre-oviposition (days)		1	2	1.6	0.5
Oviposition (days)		8	9	8.4	0.5
Longevity (days)	Female	7	9	8.2	0.8
	Male	5	7	5.9	0.9
Fecundity (eggs)		93	202	169.1	37.6
1st instar (mm)	Body length	1.9	2.1	2.0	0.1
	Cephalic area	0.3	0.3	0.3	0.0
2nd instar (mm)	Body length	4.8	5.3	4.9	0.1
	Cephalic area	0.4	0.4	0.4	0.0
3rd instar (mm)	Body length	7.4	8.4	8.0	0.4
	Cephalic area	0.7	0.7	0.7	0.0
4th instar (mm)	Body length	1.0	12.4	10.7	3.4
	Cephalic area	1.1	1.1	1.1	0.0
5th instar (mm)	Body length	13.3	15.1	14.4	0.6
	Cephalic area	1.2	1.2	1.2	0.0
Pupa (mm)	Body length	6.5	7.4	6.9	0.3
	Cephalic area	1.9	2.2	2.1	0.1
Adult (mm)	Body length	10.5	8.8	9.8	0.5
	Head width	1.2	1.0	1.1	0.1
	Wing extension	18.2	16.0	17.1	0.8

Full data described in **Supplementary Table 2**.

### 3.4 Sequencing results and phylogenetic analysis

Using ONT PCR data, a high-quality 666 bp consensus sequence for the mtCOI region was obtained, with an average coverage of approximately 3,100x. According to taxonomic classification performed using BOLD system, identified the sequence as belonging to the family Tortricidae, subfamily Tortricinae, and genus *Platynota*.

To ensure accurate classification, the 20 most similar mtCOI sequences available in BOLD were selected as references. All reference sequences corresponded to specimens within the same genus and family, confirming the taxonomic placement of our specimen (**Supplementary Table 3**). Among these, the sequence with the highest percentage of identity (99.4%) belonged to an invasive specimen reported from Manabí, Ecuador (classified as *Platynota* sp.). This high degree of genetic similarity, combined with the geographic proximity of Ecuador and the central coast of Peru, suggests a potential shared origin.

Phylogenetic analysis performed to further validate the taxonomic classification, placed our *Platynota* specimen within a well-supported clade of the tribe Sparganothidini, closely related to other species within the *Platynota* genus (92% bootstrap support value). This finding corroborates the classification by the BOLD system and confirms the placement of the specimen within the genus *Platynota*. Altogether these analyses failed to assign our specimen to the species level, suggesting it could belong to a yet undescribed species in this genus.

**Figure 4:**
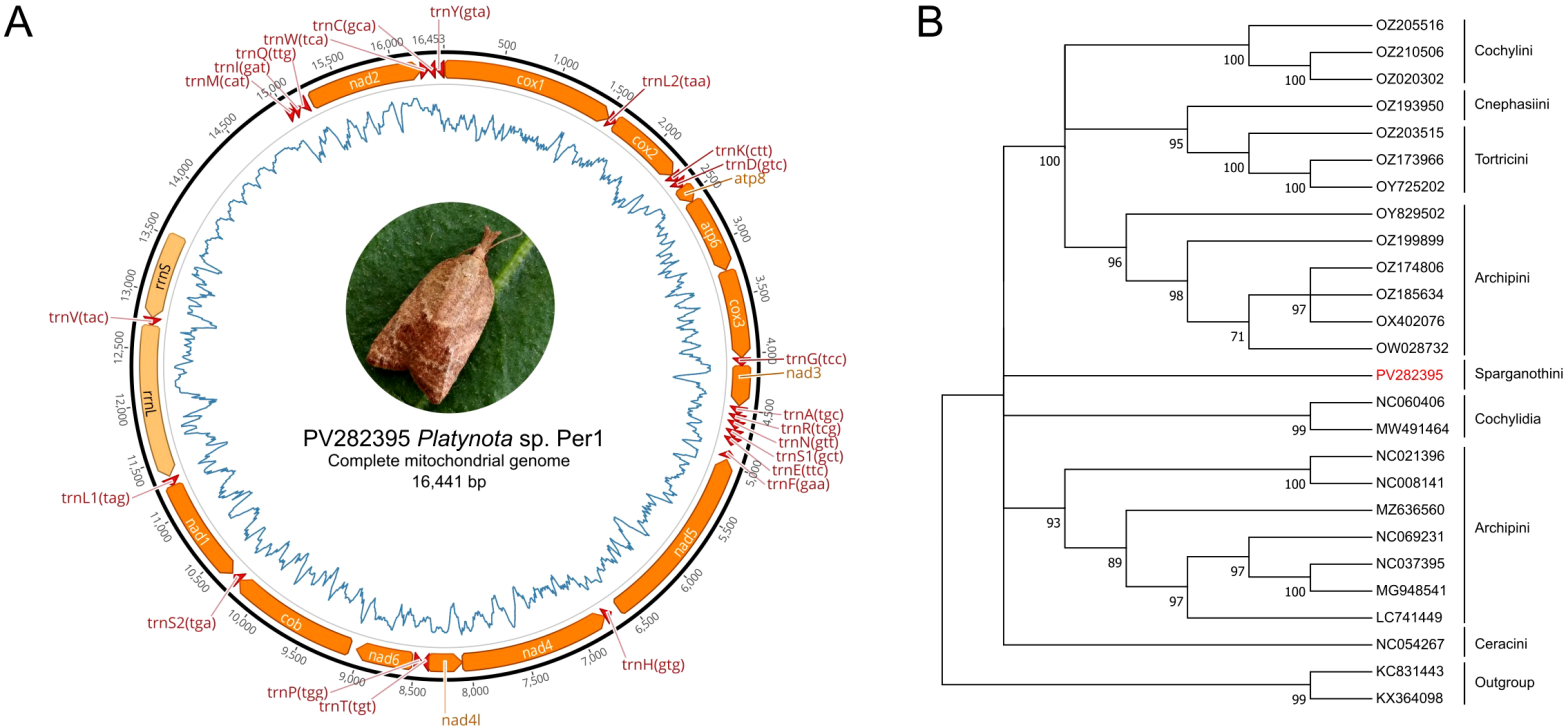
Map of the mitogenome of *Platynota* sp. Per 1 and its phylogenetic relationship with other mitogenomes of the Tortricidae family. **(A)** *Platynota* sp. Per1 mitogenome. Protein coding genes (orange) ribosomal genes (pale orange) and transfer RNA (tRNA) (red) are shown with standard abbreviations. The line inside the circle (blue) indicates the distribution of AT content. **(B)** Phylogenetic distances between mitogenomes of *Platynota* sp. Per1 and other members of the Tortricidae family. The tree was constructed using the ML method based on the GTR+G evolutionary model and a bootstrap support model based on 1,000 replicates.

The complete mitogenome of *Platynota* sp. Per1 was successfully assembled using ONT sequencing (**Figure 4A**), yielding a 16,441 bp circular, double-stranded DNA molecule with a coverage of 342x, supported by 5,634,183 of mapped reads. The mitogenome exhibited a highly skewed nucleotide composition, with adenine (A) accounting for 49.3%, thymine (T) for 33.2%, guanine (G) for 6.3%, and cytosine (C) for 11.3%, resulting in a remarkably high AT content of 82.5%. Annotation of the genome identified a total of 37 genes, including 13 protein-coding genes (PCGs). The coding sequences ranged from 165 bp (*atp8*) to 1,791 bp (*nad5*), with genes such as *cox1*, *cox2*, *cox3*, *nad3*, and *nad6 nad1*, *nad2*, *nad4*, *nad4L*, *nad5*, *atp6*, *atp8*, and *cytB* were located on the H-strand, except *nad4*, *nad4L*, and *nad5*, which were on the L-strand, 22 transfer RNA (tRNA) genes ranging in size from 56 to 71 bp genes, and two rRNA genes large with 1,349 bp and small 767 bp. Of these, 23 genes were located on the primary coding strand (J-strand or + strand), while the remaining 14 genes were positioned on the secondary coding strand (N-strand or − strand). The annotated mitogenome and associated raw sequencing data have been deposited in NCBI GenBank under accession number PV282395 (mitogenome) and SRX27742806 (SRA data), respectively.

The phylogenetic tree constructed from the complete mitogenome exhibited high robustness, with bootstrap percentages exceeding 90% in all clades, allowing clear classification by tribe (**Figure 4B**). The tribe Archipini formed two well-supported clades and had the most extensive mitogenomic data available at NCBI. In contrast, no mitogenomic information was found for the genus *Platynota* or its corresponding tribe, Sparganothidini. As a result, while *Platynota* sp. Per1 specimen belongs to the family Tortricidae, subfamily Tortricinae, its phylogenetic placement at the genus or species level remains unresolved. This high-quality mitogenome assembly represents the first complete mitogenome reported for genus *Platynota* and the tribe Sparganothini, providing a critical genomic resource for future studies on the evolutionary biology, phylogenetics, and population genetics of this genus. Additionally, it serves as a foundation for the development of molecular tools for species identification and advances our understanding of the genomic diversity within the Tortricidae family.

## 4 Discussion

In this study, we present evidence of insect damage in blueberries cultivated in Peru caused by a new tortricid pest of genus *Platynota*. The results indicate that the greatest damage of this insect on blueberry occurs during the budding stage, followed by the fruiting stage, indicating that early vegetative and reproductive stages are particularly vulnerable to tortricid pests due to the availability of tender plant tissues and their high nutrient content (36). Indeed, mature blueberry plants appear to tolerate pest infestations without significant damage, whereas younger plants can be completely defoliated (37). Additionally, reports on this crop indicate that fruit quality depends on a balance between foliage and fruit set; therefore, feeding damage caused by tortricids could negatively impact both fruit weight (grams per fruit) and overall yield (kilograms per tree) (38). This aligns with the observations made in this study (**Figure 1**). In addition, the higher damage levels recorded during the final harvest suggest that *Platynota* sp. populations may increase as the growing season progresses. Similar trends have been observed in other tortricid pests, where population densities escalate with prolonged crop availability under favorable climatic conditions (39). This highlights the importance of continuous monitoring and early intervention strategies to prevent pest outbreaks before they reach economically damaging levels (40, 41).

Wings and genitalia visual analysis enabled the identification of this blueberry pest as belonging to the family Tortricidae, genus *Platynota*. Taxonomic classification within this family relies on distinctive morphological traits, including wing shape and pattern, as well as the structure of reproductive organs, which have proven to be reliable tools for species identification (7, 12, 42) (**Figure 2**). *Platynota* species known to affect fruit crops conserve brown and ochre forewing patterns, a reticulated scale arrangement and a characteristic venation, particularly the presence of forked R3+R4 veins (39, 43). The adaptation of cryptic coloration as a defense behavior mechanism likely contributes to the persistence of infestations by minimizing predation risk, as previously observed in tortricid pests affecting fruit crops (44). Similar characteristics have been reported in *P. idaeusalis*, a pest of apple orchards in North America, which also exhibits cryptic coloration and distinct forewing markings that aid in camouflage (45). Further evidence from genitalia morphology supports the taxonomic placement of the specimens studied within the genus *Platynota*. The male genitalia, particularly the broad valvae, hook-shaped uncus, and elongated phallus (**Figure 2**), exhibit similar diagnostic traits previously described in *Platynota sultana*, a major pest of citrus and grapevines (46). Likewise, the presence of sclerotized structures in the female corpus bursae and the setose ovipositor lobes are characteristic of tortricid reproductive morphology (47). These findings reinforce the use of genitalia morphology as a reliable tool for species differentiation within Tortricidae, as highlighted in studies on *P. rostrana* and other closely related species (7).

The results confirm that *Platynota* sp. Per1, detected in Peruvian blueberry crops, exhibits a biological cycle characteristic of its genus, with an average duration of 47 ± 2.5 days. This is consistent with reported life cycles of other *Platynota* species, ranging from 38 days in *P. rostrana* to 46 days in *P. stultana*. Adult longevity varied by sex; a trait previously observed in *P. stultana* (48, 49). The average adult length (9.8 mm), aligned with the general size range of *Platynota* species, which are typically less than 12 mm (50). The pest undergoes well-defined developmental stages, with the larval phase being the longest, consisting of five instars, a pattern commonly observed in the genus. Both larval and cephalic sizes were consistent with previously reported data for *Platynota*. Regarding reproduction, fertilized females laid significantly fewer eggs (an average of 169 versus 283–308 eggs) than is reported for other *Platynota* species, although egg mass averages were similar (48, 49) (**Table 1**). It is important to note that these observations were made under laboratory conditions and may differ slightly from field conditions due to biotic factors (parasitoids, predators, pathogens) and abiotic influences (precipitation, temperature). Additionally, the life cycle of *Platynota* species may vary depending on the host plant, as observed in other studies (48, 51).

**Figure 5:**
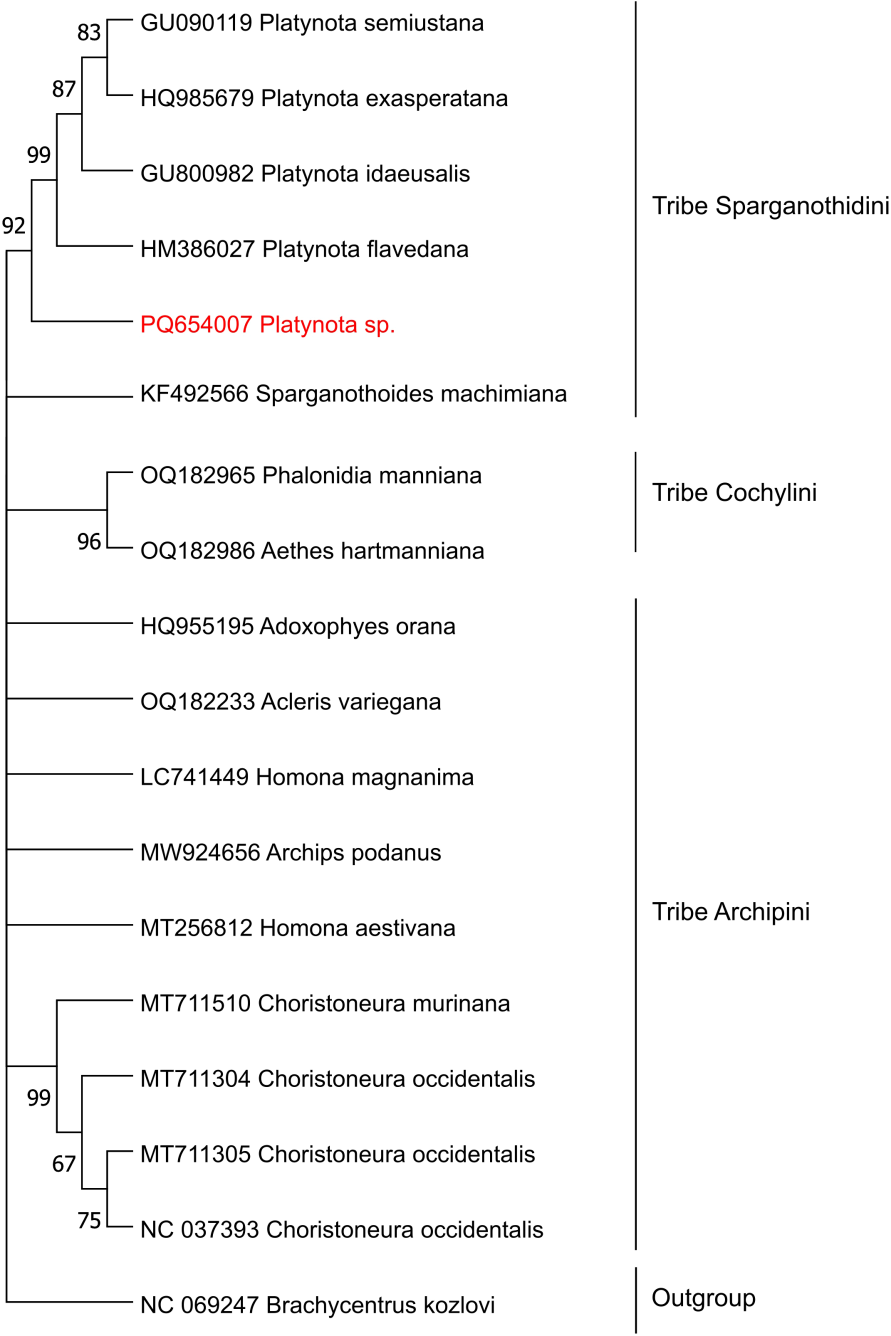
Phylogenetic tree of pests collected from blueberry crops in Peru, constructed using NJ model on mitochondrial DNA mtCOI sequences. Bootstrap values, derived from 1,000 replicates of the mtCOI region analyzed, are shown at the branch nodes. Values exceeding 50% are shown.

The use of ONT for sequencing both the COI region and the complete mitogenome from total DNA demonstrated high efficiency, achieving coverages of 3,100x and 342x, respectively. These values exceed the 100x threshold, which is widely regarded as the minimum coverage required for reliable DNA barcode analysis in laboratory settings (52–54). The mtCOI region is well established as a reliable and accurate marker for insect species identification (17, 18). Furthermore, this study demonstrates the feasibility of molecular classification without the need for nucleic acid extraction in larvae of the genus *Platynota*, offering a rapid and efficient alternative for such analyses. This approach is consistent with previous findings in *Bemisia tabaci* (24). Phylogenetic analysis using COI indicated that the studied specimen shares 92% genetic similarity with another member of the genus *Platynota* (family Tortricidae, tribe Sparganothidini) (**Figure 5**).

The sequenced mitogenome was within the size-range of other available complete mitogenomes of tortricids, ranging from 15,151 to 20,245 bp, with a typical genetic content, including 13 PCGs, 22 tRNA genes, two rRNA genes (12S and 16S), which is consistent with the ancestral pattern of Lepidoptera (21, 55). Furthermore, composition analysis indicated that the sequence was significantly biased towards adenine (A) and thymine (T), with an AT content of 82.5%, the control regions were all biased towards AT in nucleotide composition, which is consistent with most previously reported insect mitogenomes (21). The phylogenetic analysis performed at the mitochondrial level did not allow us to group our insect within any of the clades formed in the phylogenetic tree, due to the lack of available mitogenome data for members of the Tortricidae family (**Figure 4**). Currently, mitogenomic information is only available for six of the approximately 19 reported tribes (56). One of the tribes with the most information available is the Archipini tribe, which includes numerous species of global economic importance, with around 1,709 recognized species (23). mtDNA is not only a powerful tool for insect taxonomic and evolutionary classification but also plays a crucial role in insect adaptation to environmental changes, including insecticide resistance (57, 58). Several insecticides target mitochondrial functions, such as oxidative phosphorylation, disrupting energy metabolism (59). Mutations in mtDNA can alter these functions, leading to insecticide resistance by reducing the binding affinity of insecticidal compounds or enhancing metabolic detoxification pathways (60, 61). Given these implications, mitochondrial genetic data are essential for understanding resistance mechanisms and developing effective pest management strategies (62).

## 5 Conclusions

This study presents the first documented record of *Platynota* sp. (Lepidoptera: Tortricidae) as a pest of blueberry (*V. corymbosum*) in Peru, providing critical insights into its biology, life cycle, and economic impact. Our findings reveal that the larval stage inflicts the most severe damage, underscoring the importance of early detection and targeted pest management strategies to mitigate production losses.

Morphological and molecular analyses confirmed the placement of this species within the *Platynota* genus; however, species-level identification remains unresolved due to the absence of closely related reference sequences. This suggests the potential discovery of an undescribed species, highlighting the need for further taxonomic and phylogenetic studies. The sequencing and characterization of its complete mitochondrial genome provided novel insights into its genetic composition and evolutionary relationships within Tortricidae. The gene organization was consistent with other members of the family, and the high AT content aligns with typical lepidopteran mitogenomes. Notably, this study reports the first mitochondrial genome for the Sparganotidini tribe, establishing a valuable genetic resource for future comparative genomic and evolutionary research.

These findings contribute to a deeper understanding of the genetic diversity, ecological interactions, and pest potential of *Platynota* sp. in Peruvian blueberry production.

## Data Availability

The datasets presented in this study can be found in online repositories. The names of the repository/repositories and accession number(s) can be found below: Mitogenome: https://www.ncbi.nlm.nih.gov/nuccore/PV282395.1 COI: https://www.ncbi.nlm.nih.gov/nuccore/PQ654007.
